# Research on the Shearer Positioning Method Based on the MEMS Inertial Sensors/Odometer Integrated Navigation System and RTS Smoother

**DOI:** 10.3390/mi12121527

**Published:** 2021-12-08

**Authors:** Jiangtao Zheng, Sihai Li, Shiming Liu, Bofan Guan, Dong Wei, Qiangwen Fu

**Affiliations:** School of Automation, Northwestern Polytechnical University, Xi’an 710072, China; zheng_183@mail.nwpu.edu.cn (J.Z.); lisihai@nwpu.edu.cn (S.L.); liushiming000@mail.nwpu.edu.cn (S.L.); gbf1016@126.com (B.G.); weid@mail.nwpu.edu.cn (D.W.)

**Keywords:** shearer positioning, micro-electromechanical inertial measurement unit (MIMU), kalman filter, Rauch-Tung-Striebel (RTS) smoother, closing path optimal estimation model (CPOEM)

## Abstract

The shearer positioning method with an inertial measurement unit and the odometer is feasible in the longwall coal-mining process. However, the positioning accuracy will continue to decrease, especially for the micro-electromechanical inertial measurement unit (MIMU). In order to further improve the positioning accuracy of the shearer without adding other external sensors, the positioning method of the Rauch-Tung-Striebel (RTS) smoother-aided MIMU and odometer is proposed. A Kalman filter (KF) with the velocity and position measurements, which are provided by the odometer and closing path optimal estimation model (CPOEM), respectively, is established. The observability analysis is discussed to study the possible conditions under which the error states of KF can be estimated. A RTS smoother with the above-mentioned KF as the forward filter is built. Finally, the experiments of simulating the movement of the shearer through a mobile carrier were carried out, with a longitudinal movement distance of 44.6 m and a lateral advance distance of 1.2 m. The results show that the proposed method can effectively improve the positioning accuracy. In addition, the odometer scale factor and mounting angles can be estimated in real time.

## 1. Introduction

**Figure 1:**
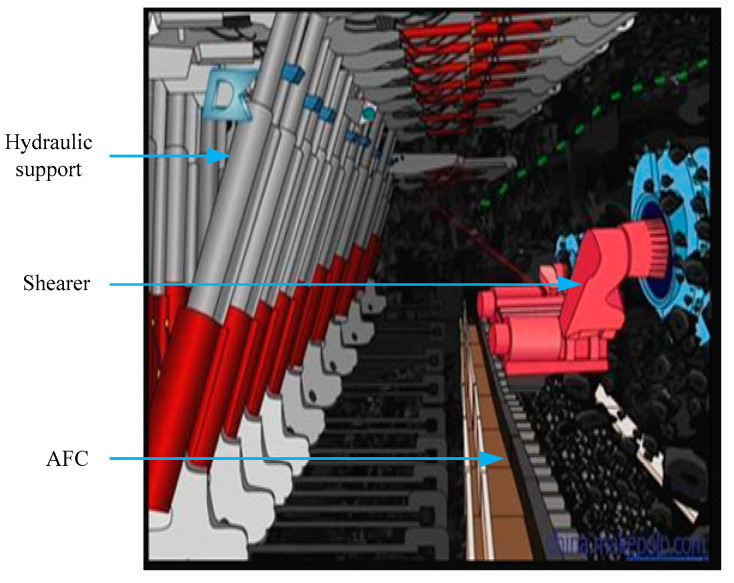
Composition of a longwall face.

Automated mining based on a longwall face has shown significant potential to improve mining productivity, increase personnel safety, and secure environmental sustain-ability [1]. As shown in Figure 1, the longwall face equipment include a shearer, some hydraulic supports, and an armored face conveyor (AFC). The shearer rides on the AFC to cut coal back and forth. The AFC provides the running track for the shearer while transporting the coal. The hydraulic supports not only support the roof but also push the AFC towards the coal seam. The position of the shearer is directly related to the control of the AFC and hydraulic supports [2]. Hence, the positioning of the shearer is the key technology to realize automated mining. The inertial measurement unit, which contains a 3D inertial sensor, is widely used to estimate the position of the shearer due to its high reliability and autonomy [3]. The micro-electromechanical inertial measurement unit (MIMU) is especially favored by the mine engineers with the advantages of low cost and small size. However, the free inertial position error can grow quickly over time due to the drifting of the inertial devices [1], which includes repeatability biases, the slow-varying drifts, and the fast-varying drifts [4,5], and thus the integrated navigation mode with MIMU as the core component becomes a better choice. The MIMU/Global Positioning System (GPS) integrated navigation system is widely used as a conventional and low-cost positioning method. Unfortunately, GPS cannot be used in underground environments. The positioning system composed by ultra-wideband range measurements can be used in GPS-denied environments [6,7]. The susceptibility of the ultra-wideband system to occlusion will affect the stability of the integrated system. The MIMU/Doppler radar sensor has been successfully applied to the positioning of a continuous miner [8]. Unlike continuous coal mining, the surrounding environment of a longwall shearer is more complicated, which is detrimental to the accuracy of the Doppler radar sensor. The above-mentioned auxiliary methods need to exchange information with the external environment, and the positioning accuracy is inevitably affected by the environment. Exploring autonomous and robust auxiliary technology has become the first problem to be solved.

Zero-velocity update (ZUPT)-aided MIMU is a simple and robust integrated strategy [9,10]. However, it requires short and frequent stops. The motion constraint-aided MIMU ZUPT method can reduce the number of stops for simple ZUPT correction [11,12], but its accuracy cannot meet the actual demand of shearer positioning. The odometer is regarded as one of the most potentially useful autonomous speed sensors for land vehicles, and the system with the MIMU and odometer has been proven to be autonomous, robust, and feasible for shearer positioning [13,14,15]. The dead reckoning (DR) with Euler angles provided by MIMU and velocity provided by the odometer is one of the methods to estimate the position of the shearer. The inertial navigation attitude error, especially the heading angle error, is the main error source precluding further improvement of DR [1]. The motion constraint [16] and closing path optimal estimation model (CPOEM) [1] are used to improve the accuracy of DR. However, these two methods can only slow down the divergence of the position error. The reason is that DR, as an open-loop structure, cannot prevent the divergence of inertial navigation attitude errors. Establishing a Kalman filter (KF) based on MIMU and the odometer is another information fusion method, which is widely used in the field of conventional land navigation [17,18,19,20]. The advantages of this method lie in the closed-loop correction of the MIMU attitude and the real-time estimation of the inertial device parameters. The effect of correction and estimation has a lot to do with the steering maneuver of the vehicle. In the case of only MIMU and the odometer, the small and slow steering maneuver of the longwall shearer is difficult to achieve the ideal positioning effect [20,21]. Therefore, we plan to build a KF based on MIMU, odometer, motion constraint, and CPOEM to achieve better positioning.

The position error of the MIMU/odometer integrated system repeats “increase–decrease” changes along with the reciprocating operation of the shearer [22], which fits the typical case of the Rauch-Tung-Striebel (RTS) smoother. RTS smoothing is a technology that uses all observation information in a certain time interval to re-estimate the state based on the KF algorithm [23]. The RTS smoothing algorithm is one of the core technologies of the position and orientation system, which can enhance the ability of the position and orientation system to be free from external disturbance [24,25,26,27]. Since the estimation accuracy of the RTS smoothing algorithm is superior to that of filtering, it is often used as a reference for post-analysis of the integrated navigation system [28,29]. In the above applications, RTS smoothing works in off-line mode, which not only requires a large storage space, but also limits its use in scenarios with real-time requirements. An on-line smoothing method can overcome the above limitations and has been successfully used in the MIMU/GPS integrated navigation system [30] and pedestrian navigation system [31]. The core idea of on-line RTS smoothing can be summarized as smoothing is executed immediately after a certain time window to achieve a near real-time application effect. Obviously, on-line RTS smoothing technology is more suitable for the needs of longwall mining. Therefore, we propose a positioning method based on MIMU, odometer, motion constraint, CPOEM, and on-line RTS smoother, whose contributions and benefits can be summarized as follows:

(1) The RTS smoother is introduced into the MIMU and odometer integrated system, which can improve the shearer positioning accuracy without additional external sensors.

(2) Observability analysis of position measurement is added to theoretically provide the estimation conditions of the main error states on the basis of the previous work on velocity measurement.

(3) The mounting angles between the MIMU frame and the odometer frame can be estimated in real time, avoiding the tedious pre-calibration process.

## 2. Mathematical Models of Velocity and Position

**Figure 2:**
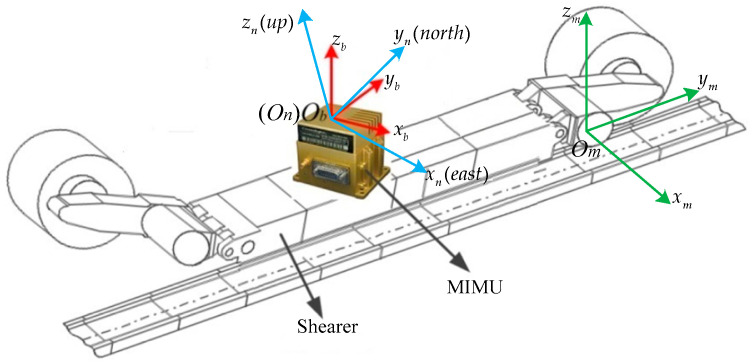
Schematic diagram of the coordinate systems.

The coordinate systems involved in this paper are shown in Figure 2, which are defined as follows: *b*-frame, the MIMU frame, which originates at the sensitive center of the MIMU, with the axes pointing to the AFC advance direction (right), shearer moving direction (forward), and upward; *m*-frame, the odometer frame, whose axes point right, forward, and upward; *n*-frame, the local-level east–north–up coordinate.

The aim of the strapdown inertial navigation system (SINS) alignment process is to determine the transition matrix from *b*-frame to *n*-frame, denoted by $C_{b}^{n}$. One of the purposes of MIMU and odometer joint calibration is to determine the mounting angles between *m*-frame and *b*-frame, which can be expressed by a vector, α. The direction cosine matrix from *b*-frame to *m*-frame is denoted by $C_{b}^{m}$.

### 2.1. Measured Velocity Model

The moving speed of the shearer is denoted by $v_{y}^{m}$, which can be measured by the odometer. Taking into account the scale factor error, $\delta k_{D}$, and measurement noise, $w_{\mathrm{OD}}$, of the odometer, the actual output, ${\tilde{v}}_{y}^{m}$, of the odometer can be expressed as [20,21,32]:

(1) $${\tilde{v}}_{y/\mathrm{OD}}^{m}=(1+\delta k_{D})v_{y}^{m}+w_{\mathrm{OD}}$$

According to the motion constraint, there is no sideslip along the AFC advance direction and no motion normal to the AFC under ideal conditions, so the velocities along the $x_{m}$ axis and $z_{m}$ axis are regarded as zero [11]. The ideal velocity of the shearer in *m*-frame can be expressed as:

(2) $$v^{m}={\left[0\;v_{y}^{m}\;0\right]}^{T}$$

Integrating (1) and (2), the measured velocity model in *m*-frame is:

(3) $${\tilde{v}}_{\mathrm{OD}/\mathrm{MC}}^{m}=(1+\delta k_{D})v^{m}+w_{\mathrm{OD}/\mathrm{MC}}$$

where $w_{\mathrm{OD}/\mathrm{MC}}$ is the noise vector, defined as:

(4) $$w_{\mathrm{OD}/\mathrm{MC}}={\left[w_{\mathrm{MC},x}\;w_{\mathrm{OD}}\;w_{\mathrm{MC},z}\right]}^{T}$$

where $w_{\mathrm{MC},x}$ and $w_{\mathrm{MC},z}$ are the motion constraint noise along the $x_{m}$ axis and the $z_{m}$ axis, respectively.

### 2.2. Measured Position Model

**Figure 3:**
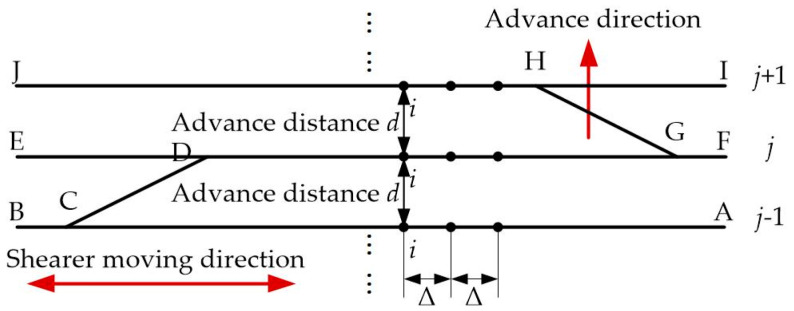
Closed path of shearer during the longwall mining.

A typical shearer operation process is to repeat “straight cutting–oblique cutting–reverse straight cutting” to form a closed path, which is shown in Figure 3 [1]. The shearer runs in the order of A–B–C–D–E–F–G–H–I–J. Assuming that the shearer moves from the right to the left of Figure 3 during the first cutting cycle, we define the end corresponding to A, F, and I as the near end of the longwall face, and the corresponding end of the B, E, and J as the far end of the longwall face. The lengths of *j* − 1, *j*, and *j* + 1 cutting cycles, corresponding to the lengths of AB, EF, and IJ, respectively, are the same. The advance distance between two adjacent cutting cycles is also the same, denoted by *d*, which can be measured by the displacement sensor fixed in the push arm of the hydraulic support. Some points with Δ interval in each cutting cycle are selected as optimal points for information fusion of KF. The ideal advance displacement of an optimal point can be expressed as:

(5) $$D_{i,j-1|j}^{m}={\left[d\;0\;0\right]}^{T},$$

where $D_{i,j-1|j}^{m}$ represents the advance displacement of point *i* from the *j* − 1 cutting cycle to the *j* cutting cycle.

According to the CPOEM principle and the longwall mining process [1], the positions of the optimal points during the next cutting cycle can be predicted through the positions of the current cutting cycle and the advance displacement. The position of point *i* in the *j* + 1 cutting cycle is expressed as:

(6) $$p_{i,j+1}=p_{i,j}+CC_{b}^{n}C_{m}^{b}D_{i,j|j+1}^{m}$$

where $p_{i,j}$ and $p_{i,j+1}$ denote the positions of point *i* in the *j* and *j* + 1 cutting cycles, respectively, which are expressed in the form of longitude, λ, latitude, L, and height, h, and matrix C, whose function is to convert the position increment in *n*-frame into the form of longitude, latitude, and height, is:

(7) $$C=\left[\begin{array}{ccc} \sec L/(R_{N}+h) & 0 & 0\\ 0 & 1/(R_{M}+h) & 0\\ 0 & 0 & 1 \end{array}\right]$$

where $R_{N}$ and $R_{M}$ denote the transverse and meridian radius of curvature, respectively, and they are the parameters used to describe the earth ellipsoid model, which are regarded as constant values in this paper.

The initial positions of the optimal points are provided in the first cutting cycle. The specific process is as follows:

- The two optimal points corresponding to both ends of the longwall face can be accurately measured in advance, as mentioned in [16,33].
- To avoid additional surveying and mapping, the initial positions of the remaining optimal points can be obtained using the position estimates of the integrated system in the first cutting cycle. This initial value assignment method is mentioned in [1].

Taking into account the existence of measurement noise, the measured position, ${\tilde{p}}_{i/\mathrm{CPOEM}}$, of point *i* predicted by CPOEM can be approximated as:

(8) $${\tilde{p}}_{i/\mathrm{CPOEM}}\approx p_{i}+w_{i/\mathrm{CPOEM}}$$

where $w_{i/\mathrm{CPOEM}}$ is the CPOEM noise vector of point *i*.

## 3. Integrated Navigation and RTS Smoother Models

### 3.1. Error State Equation of Integrated Navigation System

The mounting angles between *m*-frame and *b*-frame are inevitable, even if the pre-calibration process is carried out. The residual installation errors after calibration can be regarded as random constants, denoted by $\delta \alpha ={\left[\delta \alpha_{x}\;\delta \alpha_{y}\;\delta \alpha_{z}\right]}^{T}$. The scale factor error of the odometer can also be regarded as a random constant. Thus, the following equation can be obtained:

(9) $$\{\begin{array}{c} \delta \dot{\alpha }=0_{3\times 1}\\ \delta {\dot{k}}_{D}=0 \end{array}$$

where $0_{a\times b}$ is a a×b zero matrix.

The direction cosine matrix, ${\tilde{C}}_{b}^{m}$, with the residual error δα satisfies:

(10) $${\tilde{C}}_{b}^{m}=C_{b}^{m}[I+(\delta \alpha \times )]$$

where I is a third-order unit matrix and (δα×) denotes the skew symmetric matrix of δα with 3 rows and 3 columns.

When the mounting angles, α, are controlled within a small range by a precise mechanical installation, the equation ${\tilde{C}}_{b}^{m}=I$ can be obtained directly without the need to perform the pre-calibration process.

Some related error state vectors of the SINS satisfy the following equation:

(11) $$\{\begin{array}{c} {\tilde{v}}_{\mathrm{SIN}S}^{n}=v^{n}+\delta v^{n}\\ {\tilde{C}}_{n}^{b}=C_{n}^{b}[I+(\varphi^{n}\times )]\\ {\tilde{p}}_{\mathrm{SIN}S}=p+\delta p \end{array}$$

where $v^{n}$, $C_{n}^{b}$, and p are the true velocity, attitude matrix, and position, respectively, and ${\tilde{v}}_{\mathrm{SIN}S}^{n}$, ${\tilde{C}}_{n}^{b}$, and ${\tilde{p}}_{\mathrm{SIN}S}$ are the error-contaminated velocity, attitude matrix, and position, respectively, calculated by the SINS. $\delta v^{n}={\left[\delta v_{E}\;\delta v_{N}\;\delta v_{U}\right]}^{T}$ and δp are the velocity errors and position errors of the SINS, respectively, $\varphi^{n}={\left[\varphi_{E}\;\varphi_{N}\;\varphi_{U}\right]}^{T}$ is the misalignment angles of ${\tilde{C}}_{n}^{b}$ in *n*-frame, and the subscripts E, N, and U are the east, north, and up directions in *n*-frame.

A 19-dimensional error state vector is defined as:

(12) $$x(t)={\left[{\left(\delta v^{n}\right)}^{T}\;{\left(\varphi^{n}\right)}^{T}\;{\left(\delta p\right)}^{T}\;{\left(\varepsilon^{b}\right)}^{T}\;{\left(\nabla^{b}\right)}^{T}\;{\left(\delta \alpha \right)}^{T}\;\delta k_{D}\right]}^{T}$$

where $\varepsilon^{b}$ and $\nabla^{b}$ are the gyro and accelerometer biases, respectively.

Taking into account the low-speed motion characteristics of the shearer, the velocity-related terms in the SINS error equation can be ignored. Then, the SINS error equation can be simplified to:

(13) $$\{\begin{array}{c} \begin{array}{c} \delta {\dot{v}}^{n}=-2\omega_{ie}^{n}\times \delta v^{n}+f^{n}\times \varphi^{n}+C_{b}^{n}\nabla^{b}\\ {\dot{\varphi }}^{n}=-\omega_{ie}^{n}\times \varphi^{n}-C_{b}^{n}\varepsilon^{b} \end{array}\\ \delta \dot{p}=C\delta v^{n}\\ \begin{array}{c} {\dot{\varepsilon }}^{b}=0_{3\times 1}\\ {\dot{\nabla }}^{b}=0_{3\times 1} \end{array} \end{array}$$

where $\omega_{ie}^{n}$ denotes the rotation rate vector of the earth and $f^{n}=C_{b}^{n}f^{b}$, in which $f^{b}$ is the specific force measured by the accelerometers.

The error state equation of the integrated navigation system can be expressed as:

(14) $$\dot{x}(t)=\underset{F(t)}{\underset{︸}{\left[\begin{array}{cc} F_{\mathrm{SIN}S} & 0_{15\times 4}\\ 0_{4\times 15} & 0_{4\times 4} \end{array}\right]}}x(t)+w(t)$$

where w(t) is the noise vector of the integrated system and the 15 × 15 transition matrix $F_{\mathrm{SIN}S}$ is denoted by:

(15) $$F_{\mathrm{SIN}S}=\left[\begin{array}{ccccc} -2(\omega_{ie}^{n}\times ) & \left(f^{n}\times \right) & 0_{3\times 3} & 0_{3\times 3} & C_{b}^{n}\\ 0_{3\times 3} & -(\omega_{ie}^{n}\times ) & 0_{3\times 3} & -C_{b}^{n} & 0_{3\times 3}\\ C & 0_{3\times 3} & 0_{3\times 3} & 0_{3\times 3} & 0_{3\times 3}\\ 0_{6\times 3} & 0_{6\times 3} & 0_{6\times 3} & 0_{6\times 3} & 0_{6\times 3} \end{array}\right]$$

The discretized error state equation corresponding to (14) is:

(16) $$X_{k}=\Phi_{k,k-1}X_{k-1}+W_{k-1},$$

where $X_{k}$ and $X_{k-1}$ are the discretized error state vectors at $t_{k}$ and $t_{k-1}$, respectively, $W_{k-1}$ denotes the system noise matrix at $t_{k-1}$, and $\Phi_{k,k-1}$ is the discretized state transition matrix that satisfies the following equation:

(17) $$\Phi_{k,k-1}{=e}^{TF(t_{k-1})}$$

where T denotes the filtering period.

### 3.2. Measurement Equations of Velocity and Position

The velocity, ${\tilde{v}}_{\mathrm{SIN}S}^{m}$, in *m*-frame calculated by the SINS can be expressed as:

(18) $$\begin{array}{lll} {\tilde{v}}_{\mathrm{SIN}S}^{m} & = & {\tilde{C}}_{b}^{m}{\tilde{C}}_{n}^{b}{\tilde{v}}_{\mathrm{SIN}S}^{n}\\ & \approx & v^{m}+C_{b}^{m}C_{n}^{b}\delta v^{n}-C_{b}^{m}C_{n}^{b}(v^{n}\times )\varphi^{n}\\ & & -C_{b}^{m}(v^{b}\times )\delta \alpha \end{array}$$

where $v^{b}=C_{n}^{b}v^{n}$.

We find the difference between (3) and (18) to obtain the velocity measurement equation as:

(19) $$z_{v}={\tilde{v}}_{\mathrm{SIN}S}^{m}-{\tilde{v}}_{\mathrm{OD}/\mathrm{MC}}^{m}=H_{v}x(t)+w_{\mathrm{OD}/\mathrm{MC}}$$

where the measurement matrix, $H_{v}$, of velocity is expressed as:

(20) $$H_{v}=\left[\begin{array}{ccc} \begin{array}{cc} \begin{array}{cc} C_{b}^{m}C_{n}^{b} & -C_{b}^{m}C_{n}^{b}(v^{n}\times ) \end{array} & 0_{3\times 9} \end{array} & -C_{b}^{m}(v^{b}\times ) & -v^{m} \end{array}\right]$$

Similarly, the position measurement equation can be expressed as:

(21) $$z_{p}={\tilde{p}}_{\mathrm{SIN}S}-{\tilde{p}}_{\mathrm{CPOEM}}=H_{p}x(t)+w_{\mathrm{CPOEM}}$$

where the measurement matrix, $H_{p}$, of the position is given by:

(22) $$H_{p}=\left[\begin{array}{ccc} 0_{3\times 6} & I & 0_{3\times 10} \end{array}\right]$$

The update of the position measurement depends on the mileage calculated by the odometer, which means that the update period of the position measurement is an integer multiple of that of the velocity measurement. Therefore, the overall measurement model includes two forms: simultaneous velocity and position measurement update and separate velocity measurement update. The specific update conditions and model equations are as follows.

If the shearer is not in the first cutting cycle and the position measurement is judged to be valid by the mileage calculated by the odometer, then:

(23) $$\{\begin{array}{c} z=\left[\begin{array}{c} z_{v}\\ z_{p} \end{array}\right]\\ H=\left[\begin{array}{c} H_{v}\\ H_{p} \end{array}\right] \end{array}$$

where z and H, respectively, represent the overall measurement vector and measurement matrix.

Otherwise:

(24) $$\{\begin{array}{c} z=z_{v}\\ H=H_{v} \end{array}$$

The discretized measurement equation is:

(25) $$Z_{k}=H_{k}X_{k}+V_{k}$$

where $Z_{k}$, $H_{k}$, and $V_{k}$ are the discretized measurement vector, the discretized measurement matrix, and the measurement noise sequence at $t_{k}$, respectively.

### 3.3. Observability Analysis of Integrated System

The main purpose of observability analysis is to study the observability and estimatable conditions of the error state. An observability analysis method directly relies on the state and measurement equations to investigate the observability.

#### 3.3.1. Observability Analysis Based on the Velocity Measurement

The observability analysis based on the velocity measurement has been discussed in detail in our previous work [34], so here, we directly summarize the observability conclusions about the velocity measurement with the motion characteristics of the longwall shearer.

- The velocity errors, $\delta v^{n}$, are observable, and the estimation accuracy is related to the estimation degree of other error states.
- The position errors, δp, are unobservable, but the estimation accuracy will still be improved with the effective estimation of the velocity errors.
- The acceleration and deceleration process of the shearer is the premise of exciting the error states $\delta \alpha_{x}$, $\delta \alpha_{z}$, and $\delta k_{D}$, which contribute to the positioning errors. The error $\delta \alpha_{y}$ is unobservable.
- The error $\nabla_{z}^{b}$ is observable. The separation of $\nabla_{x}^{b}$ and $\nabla_{y}^{b}$, and the distinction of $\varepsilon_{x}^{b}$ and $\varepsilon_{y}^{b}$, improving the estimation accuracy of $\varphi^{n}$, depend on the turning motion of the shearer. The azimuth error, $\varphi_{U}$, is directly related to the lateral positioning error, thus, restricting the estimation accuracy of error $\delta \alpha_{z}$.

It can be concluded that frequent turning of the shearer is necessary to improve the estimation accuracy of the error states. However, limited by working conditions, the longwall shearer has very few steering maneuvers. Therefore, it is difficult to achieve high shearer positioning accuracy using only the integrated system of MIMU and the odometer.

#### 3.3.2. Observability Analysis Based on the Position Measurement

In (21), the measurement values are constructed by the position errors. Therefore, the position errors, δp, are observable. The estimation accuracy of δp depends on the position accuracy of the optimal points predicted by CPOEM. The vectors that make up the measurement values have the same initial position errors, so the initial position errors cannot be estimated.

Taking the time derivative of (21) obtains:

(26) $${\dot{z}}_{p}\approx C\delta v^{n}$$

Equation (26) indicates that the velocity errors, $\delta v^{n}$, are observable, and the estimation accuracy is determined by the position errors.

The terms related to $v^{n}$ in the mathematical models can be ignored due to the low-speed characteristics of the shearer. Therefore, the time derivative of (26) can be simplified as:

(27) $$\begin{array}{l} (C^{-1}){\ddot{z}}_{p}\approx (f^{n}\times )\varphi^{n}-2(\omega_{ie}^{n}\times )\delta v^{n}+\nabla^{n}\\ =\left[\begin{array}{c} -f_{U}\varphi_{N}+f_{N}\varphi_{U}+2\omega_{ie}\sin L\delta v_{N}-2\omega_{ie}\cos L\delta v_{U}+\nabla_{E}\\ f_{U}\varphi_{E}-f_{E}\varphi_{U}-2\omega_{ie}\sin L\delta v_{E}+\nabla_{N}\\ -f_{N}\varphi_{E}+f_{E}\varphi_{N}+2\omega_{ie}\cos L\delta v_{E}+\nabla_{U} \end{array}\right] \end{array}$$

where $f^{n}={\left[f_{E}\;f_{N}\;f_{U}\right]}^{T}$, $\nabla^{n}={\left[\nabla_{E}\;\nabla_{N}\;\nabla_{U}\right]}^{T}$, and $\omega_{ie}$ is the rotation rate of the earth.

The acceleration and deceleration process of the shearer makes at least one of $f_{E}$ and $f_{N}$ non-zero, which means that the coefficient of $\varphi_{U}$ will not be 0 in (27). That is to say, the acceleration and deceleration process can improve the observability of the azimuth error, $\varphi_{U}$.

Let us assume a high-precision position predicted by CPOEM is obtained. Then, the errors δp and $\delta v^{n}$ will be accurately estimated. The estimation accuracy of the azimuth error, $\varphi_{U}$, depends on the separating degree from the errors $\varphi_{E}$, $\varphi_{N}$, $\nabla_{E}$, and $\nabla_{N}$. Although the state errors $\delta k_{D}$, $\delta \alpha_{x}$, and $\delta \alpha_{z}$ are not directly observable in (21), (26), and (27), their accuracy will be improved with the estimation of error states δp and $\varphi^{n}$. Similarly, the estimation accuracy of $\varepsilon^{b}$ will also be improved with the estimation of other state errors.

### 3.4. RTS Smoothing

RTS smoothing uses all the measurement values obtained in a time interval to estimate the error states at every epoch in this interval. A typical RTS smoothing process is divided into two steps: forward filtering and backward smoothing.

The forward filtering is obtained through standard KF, whose basic equation for discretization can be expressed as:

(28) $$\{\begin{array}{c} \begin{array}{c} X_{k,k-1}=\Phi_{k,k-1}X_{k-1}\\ P_{k,k-1}=\Phi_{k,k-1}P_{k-1}\Phi_{k,k-1}^{T}+Q_{k-1} \end{array}\\ K_{k}=P_{k}H_{k}^{T}R_{k}^{-1}\\ \begin{array}{c} X_{k}=X_{k,k-1}+K_{k}(Z_{k}-H_{k}X_{k,k-1})\\ P_{k}=(I-K_{k}H_{k})P_{k,k-1} \end{array} \end{array}$$

where $X_{k,k-1}$ and $P_{k,k-1}$ are the one-step predicted states and covariance at $t_{k}$, calculated from the information at $t_{k-1}$, $P_{k-1}$ and $P_{k}$ denote the state estimate covariance at $t_{k-1}$ and $t_{k}$, respectively, and $Q_{k-1}$ is the variance matrix of the system noise sequence $W_{k-1}$ at $t_{k-1}$. $K_{k}$ represents the filter gain matrix at $t_{k}$, and $R_{k}$ is the variance matrix of the measurement noise sequence $V_{k}$ at $t_{k}$.

The forward filtering in this paper refers to the KF based on the velocity and position measurements mentioned above. In the forward filtering process, it is required to save $X_{k,k-1}$, $X_{k}$, $\Phi_{k,k-1}$, $P_{k,k-1}$, and $P_{k}$ at every epoch in the time interval. After completing the forward filtering in the time interval, the backward smoothing is performed. The procedure of the backward smoothing is broken down into the following steps:

1. Backward Smoothing Initialization

Define the time interval as [$t_{j}$,$t_{j+N}$], then the initialization equation is given as:

(29) $$\{\begin{array}{c} X_{\mathrm{rts},j+N}=X_{j+N}\\ P_{\mathrm{rts},j+N}=P_{j+N} \end{array},$$

where $X_{j+N}$ and $P_{j+N}$ are the error state vector and the mean square error matrix of the forward filtering process at $t_{j+N}$, and $X_{\mathrm{rts},j+N}$ and $P_{\mathrm{rts},j+N}$, which are the initial values required for backward smoothing, denote the error state vector and the mean square error matrix of the backward smoothing process at $t_{j+N}$.

2. Backward Smoothing Update

The recursive equations of the backward smoothing update are given by:

(30) $$\{\begin{array}{c} X_{\mathrm{rts},k}=X_{k}+A_{k}(X_{rts,k+1}-X_{k+1,k})\\ P_{\mathrm{rts},k}=P_{k}+A_{k}(P_{rts,k+1}-P_{k+1,k})A_{k}^{T} \end{array},$$

where $A_{k}$ is the smoother gain, determined as:

(31) $$A_{k}=P_{k}\Phi_{k+1,k}^{T}P_{k+1,k}^{-1},$$

**Figure 4:**
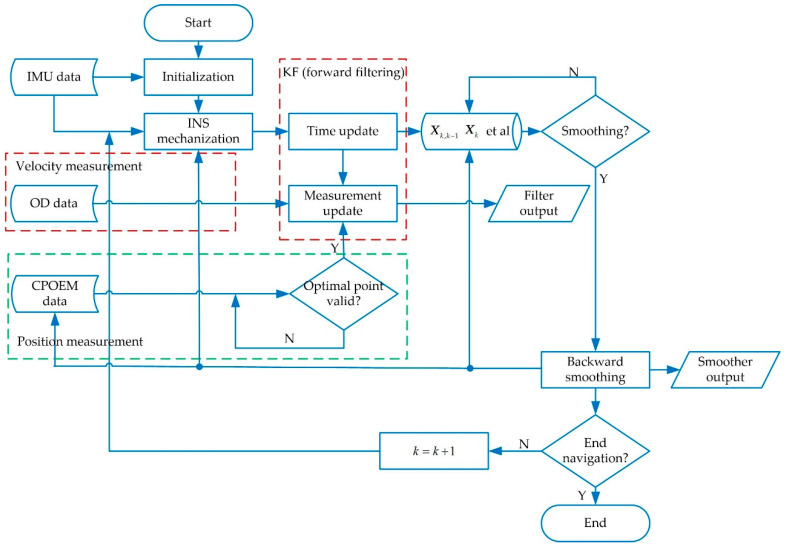
Flowchart of KF and the RTS smoother.

Sorting out the process of KF and the RTS smoother, the flowchart is shown in Figure 4. If mod(*S*, Δ) = = 0, where *S* denotes the mileage of the shearer calculated by the odometer, the optimal point from the CPOEM data is valid and the position measurement update of KF is performed. Backward smoothing is only executed when the shearer is at both ends of the longwall face and not in the first cutting cycle. The positions of the optimal points of the next cycle will be updated immediately after the end of backward smoothing.

## 4. Experiments

**Figure 5:**
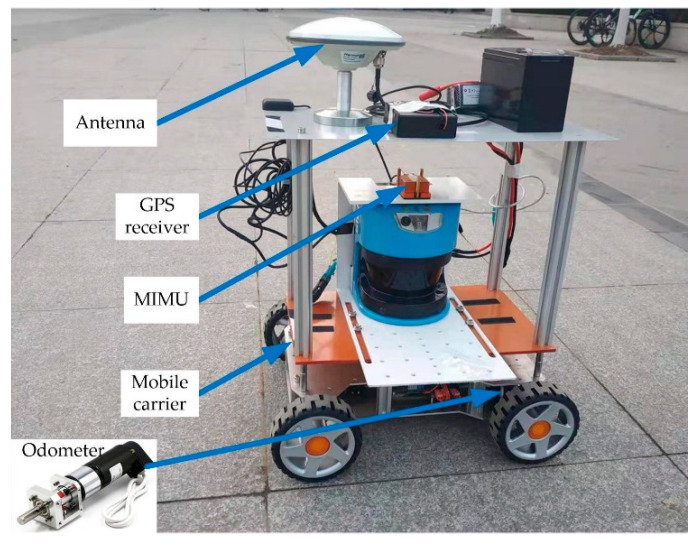
Experiment mobile carrier and the equipment.

To evaluate the performance of the proposed positioning method, experiments were carried out, as shown in Figure 5. A mobile carrier equipped with the MIMU (Xsens MTi-G-700) and the odometer simulated the movement of the shearer. The MIMU was installed on the mobile carrier through an adapter plate and the odometer was connected to its wheel. A GPS receiver with an antenna was also installed on the mobile carrier. The GPS receiver can output centimeter-level positioning results through the network differential technology. The positioning result of the GPS receiver only provides an evaluation basis for tests. The specifications of the MIMU and the initial errors are listed in Table 1, which are related to the initial parameter configuration of the Kalman filter. The specifications and the initial attitude error refers to the MTI user manual [35]. The initial position error refers to the network differential positioning accuracy [36]. The initial parameters of the filter are set as Appendix A.

**Figure 6:**
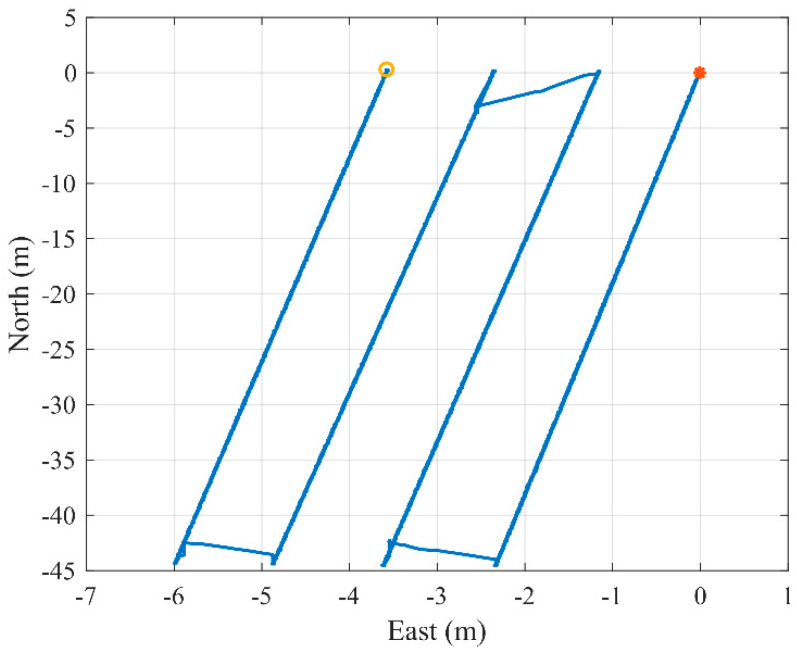
Trajectory of the mobile carrier during the experiment.

The mobile carrier simulated four cutting processes of the shearer with a reciprocating travel distance of 44.6 m and an advance distance of 1.2 m, as shown in Figure 6. Figure 6 is drawn by the network differential results provided by the GPS receiver. The symbols “*” and “o” denote the start and end of the trajectory, respectively.

**Figure 7:**
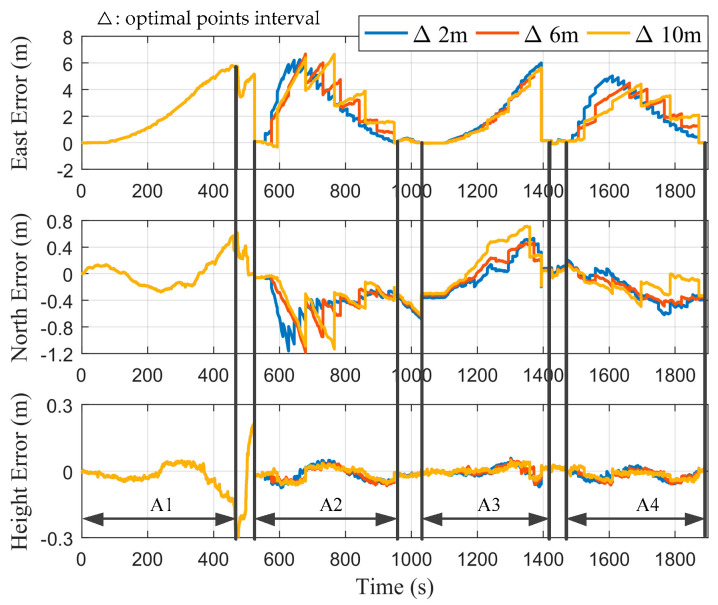
Positioning errors without performing RTS smoothing under different values of Δ.

**Figure 8:**
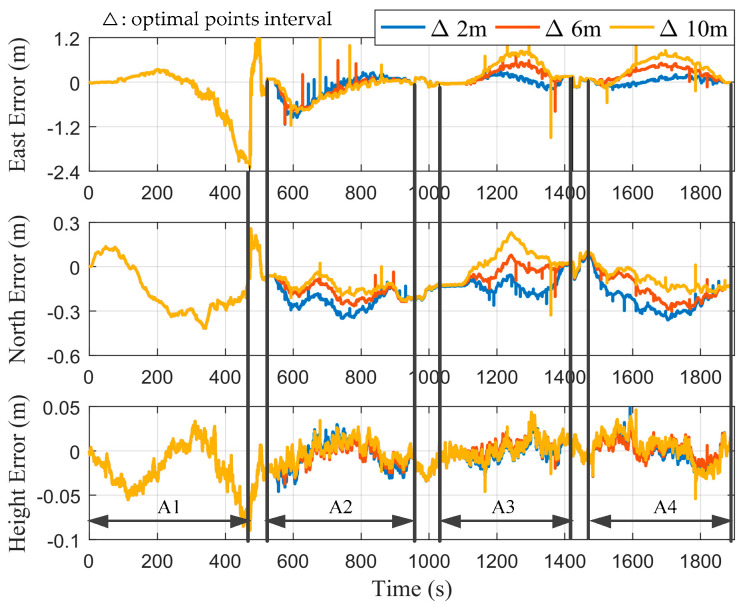
Positioning errors with performing RTS smoothing under different values of Δ.

In order to study the influence of the selection of optimal points on the estimation of error states, we set the optimal points interval, Δ, as 2, 6, and 10 m, in turn. Figure 7 shows the positioning errors of the proposed integrated system without performing RTS smoothing. A1–A4 corresponded to the time periods of the first to fourth cutting cycles, respectively. The remaining time periods corresponded to the process of the shearer processing the end coal seam. It can be seen that during the first cutting cycle, the curves of different values of Δ overlapped in the east, north, and height directions, respectively, since position measurement filtering was not performed. During the second to fourth cutting cycles, the curves of the east and north errors were smoother as Δ decreased. Figure 8 shows the positioning errors of the proposed integrated system with performing RTS smoothing. It was straightforward to see that the positioning accuracy of all three axes with performing RTS smoothing was higher than that of not performing, regardless of the value of Δ. In addition, it can be seen that there were larger burrs in the east and north directions as Δ increased. Therefore, we can choose a smaller value of Δ to obtain smoother position estimations.

**Figure 9:**
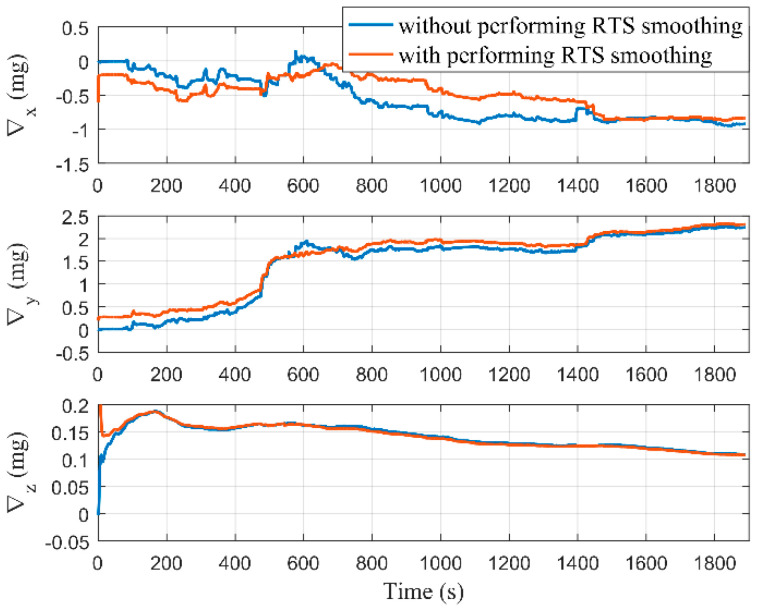
Estimation results of the accelerometer biases with and without performing RTS smoothing.

**Figure 10:**
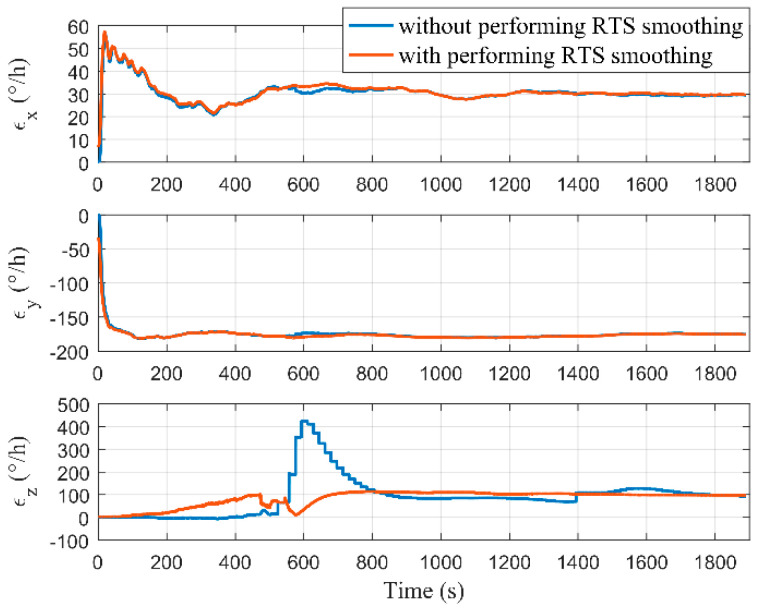
Estimation results of the gyro biases with and without performing RTS smoothing.

**Figure 11:**
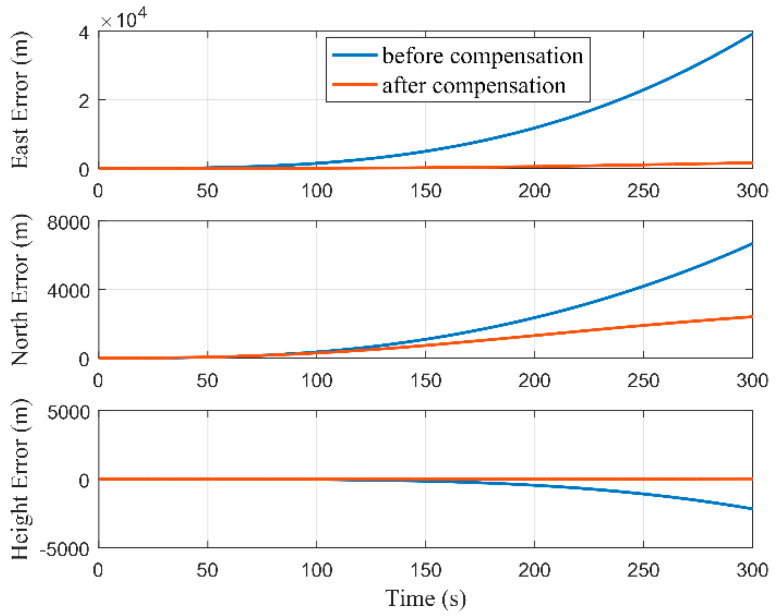
Positioning errors of pure navigation calculations before and after compensation of the MIMU biases.

Figure 9 and Figure 10, respectively, show the estimations of the accelerometer and gyro biases with and without performing RTS smoothing, while we set Δ as 2 m. It can be seen that the estimations of MIMU biases were equivalent with and without performing RTS smoothing when the estimators tended to be stable. Although the true values of MIMU biases cannot be accurately obtained, the results of the pure navigation before and after the bias compensation can reflect their estimation accuracy. We used the first 300 s of data to perform the pure navigation calculations before and after the bias compensation, and the results are shown in Figure 11. Compared with the results before compensation of the MIMU biases, the positioning accuracy was greatly improved after compensation. Therefore, the MIMU biases can be effectively estimated. The RTS smoothing technology only reduced the error fluctuations in the estimation process, and did not affect the final estimation accuracy.

**Figure 12:**
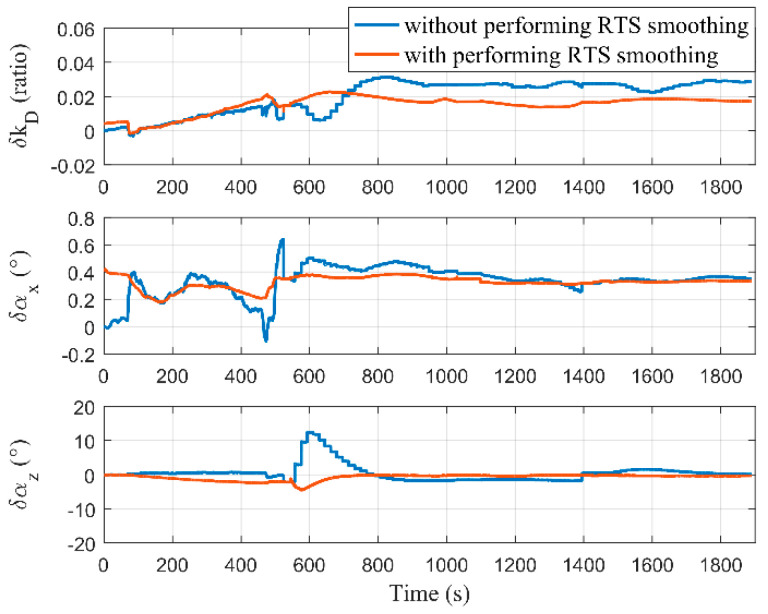
Estimation results of the odometer scale factor and mounting angles with and without performing RTS smoothing.

**Figure 13:**
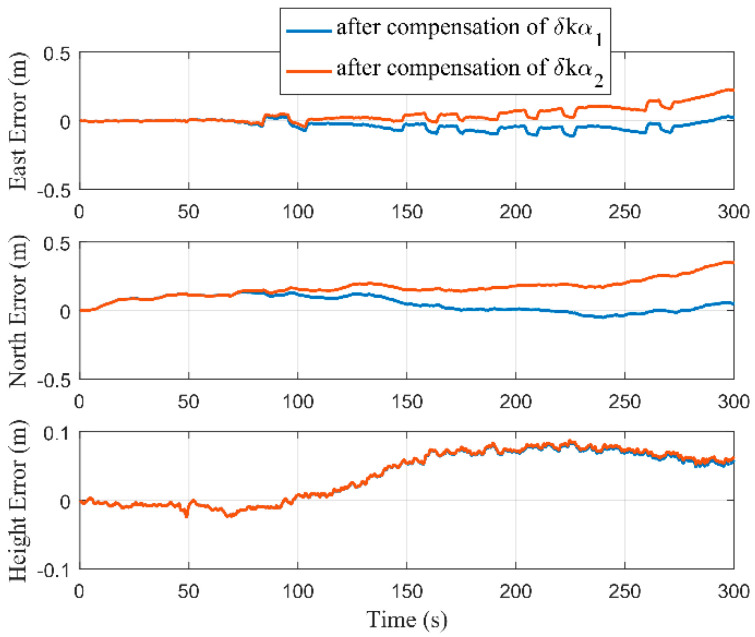
Positioning errors of DR calculations after compensation of $\delta k\alpha_{1}$ and $\delta k\alpha_{2}$.

The estimation results of the odometer scale factor and mounting angles are shown in Figure 12. We know that $\delta \alpha_{y}$ is always unobservable from the observability analysis. Therefore, its estimation curve is not drawn in this paper. The estimated scale factor and mounting angles, whose record order was $\delta k\alpha ={\left[\delta k_{D}\;\delta \alpha_{X}\;\delta \alpha_{z}\right]}^{T}$, with and without performing RTS smoothing, were $\delta k\alpha_{1}={\left[0.017\;0.333{}^{\circ}-0.307{}^{\circ}\right]}^{T}$ and $\delta k\alpha_{2}={\left[0.029\;0.352{}^{\circ}\;0.093{}^{\circ}\right]}^{T}$, respectively. The positioning accuracy of the DR algorithm is restricted by δkα. In other words, the DR results can reflect the estimation effect of these three error states. The DR navigation calculations were performed after the compensation of $\delta k\alpha_{1}$ and $\delta k\alpha_{2}$ using the data that has been compensated for the MIMU biases, and the results are shown in Figure 13. The horizontal positioning accuracy after compensation of $\delta k\alpha_{1}$ was better than that after compensation of $\delta k\alpha_{2}$, which means that the estimation accuracy of the error states $\delta k_{D}\;$ and $\delta \alpha_{z}$ with performing RTS smoothing was improved. According to the theory of observability analysis, the estimation accuracy of error states (such as $\delta k_{D}\;$ and $\delta \alpha_{z}$) can be improved by feedback when the position errors of the optimal points were reduced. Since the positioning accuracy with performing the RTS smoothing was better than that without performing (shown in Figure 7 and Figure 8), the position errors of the corresponding optimal points were also smaller. Therefore, the results of Figure 13 are consistent with the observability analysis.

The shearer is a long and narrow machine with limited space for the installation of external sensors. The small size of MEMS inertial sensors makes them a goal pursued by researchers. However, small-sized and low-cost inertial sensors often have low measurement accuracy. Therefore, we studied the impact of MIMU, which has a lower accuracy than MTi-G-700, on positioning accuracy of the mobile carrier. The idea of MIMU data generation is to superimpose errors on the original gyroscope and accelerometer data corresponding to Figure 6 to simulate lower-precision MEMS inertial sensor data. The error components of the gyroscope and accelerometer include repeatability biases, slow-varying drifts, and fast-varying drifts [4,5]. The repeatability biases can be regarded as a random constant. The slow-varying drift can be approximated as white noise due to the short correlation time of MIMU. The fast-varying drift is often abstracted as a white noise process. The white noise process is usually evaluated by random walk. In summary, the error model of the gyroscope and accelerometer can be expressed as:

(32) $$\{\begin{array}{c} \varepsilon_{s}^{b}=\varepsilon^{b}+\varepsilon_{w}^{b}\\ \nabla_{s}^{b}=\nabla^{b}+\nabla_{w}^{b} \end{array}$$

where $\varepsilon_{s}^{b}$ and $\nabla_{s}^{b}$ represent the total gyroscope and accelerometer errors, respectively, $\varepsilon^{b}$ and $\nabla^{b}$ denote the constant biases of the gyroscope and accelerometer, respectively, and $\varepsilon_{w}^{b}$ and $\nabla_{w}^{b}$ are the random walk noises of the gyroscope and accelerometer, respectively.

**Figure 14:**
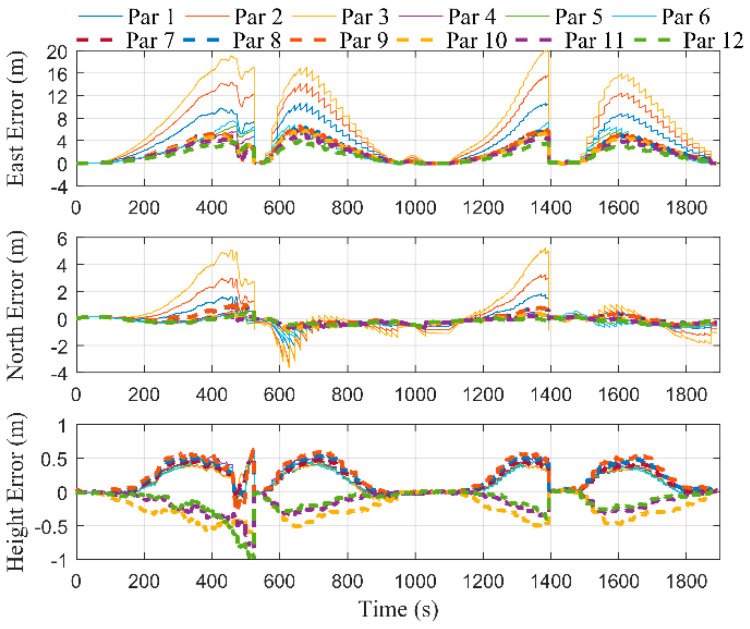
Positioning errors without performing RTS smoothing under different error parameters.

**Figure 15:**
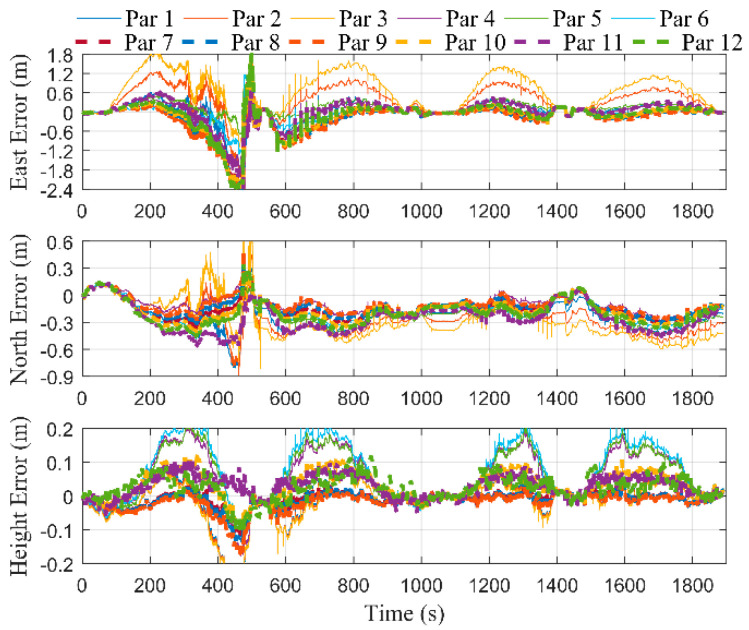
Positioning errors with performing RTS smoothing under different error parameters.

A series of MIMU errors were designed, as listed in Table 2. The errors in Table 2 were added to the original data corresponding to Figure 6. The position errors before and after RTS smoothing are shown in Figure 14 and Figure 15, respectively. Similar to the phenomenon in Figure 7 and Figure 8, even if the accuracy of the inertial sensors is reduced, the positioning accuracy can still be improved after performing RTS smoothing. It can be seen from Figure 15 that the east errors of Par 3 and Par 2 are much larger than those of other parameters, and the east error of Par 3 is greater than that of Par 2. This is because the constant biases of the gyroscope are not fully estimated, and the residual constant component is larger as the constant biases increase. Correspondingly, the attitude and position errors are bound to be greater. In summary, it can be concluded that the proposed method can still improve the positioning accuracy even if the low-precision inertial sensors are used, and the positioning accuracy is closely related to the sensor parameters, especially the gyroscope.

**Figure 16:**
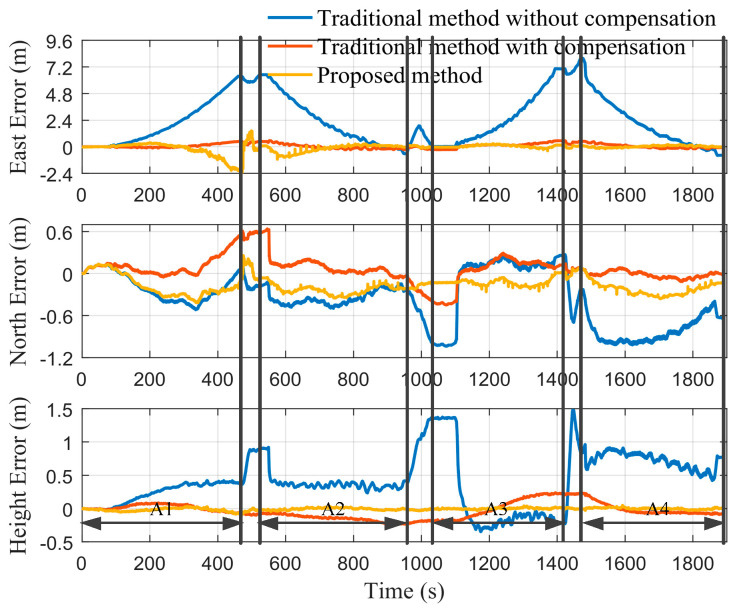
Comparison of positioning errors between the proposed method and the traditional method.

In order to further verify the performance of the method proposed in this paper, it is compared with the traditional method. The method mentioned in [1] is state-of-the-art based on the SINS and the odometer. However, this method does not have the ability to autonomously estimate the biases of inertial sensors, mounting angles between MIMU and the odometer, and the scale factor error of the odometer. Considering that the above parameters have a great influence on the traditional method, it was compensated with the parameter values obtained by the proposed method. The positioning results of the traditional method before and after the estimated parameter compensation and the method proposed in this paper are shown in Figure 16. It can be seen from Figure 16 that after the parameters are compensated, the positioning errors of the traditional method are significantly reduced. It can also be seen that the accuracy of the method proposed in this paper is better than the traditional method. In order to visually describe the accuracy improvement range of the proposed method, the spherical error probable (SEP) is calculated. The SEP is a universal evaluation method of 3D positioning accuracy [37], which is listed in Table 3. A1–A4 correspond to the first to fourth cutting cycles, respectively. It can be seen from Table 3 that compared with the traditional method without compensation, the positioning accuracy of the proposed method increases by 71.43%, 83.92%, 94.30% and 92.64% in turn from A1 to A4. Since the position measurement is not performed in A1, and the optimal point position of A2 is related to that of A1, the SEP of the proposed method is larger in A1 and A2. It is significantly reduced after A3. Compared with the traditional method with compensation, the positioning accuracy of the proposed method increases by 60.61% in A3 and is equivalent in A4. The above phenomenon not only proves the superiority of the proposed method in positioning accuracy, but also further shows that the method proposed in this paper can effectively estimate some parameters, such as biases of the MIMU, odometer scale factor, etc.

## 5. Conclusions

This paper proposed a positioning method of the shearer based on an integrated system and RTS smoothing technology. Performing RTS smoothing on the basis of the Kalman filter is a major feature of this paper. An experiment was carried out to verify the performance of the proposed positioning method. The experimental results showed that the positioning accuracy after performing RTS smoothing was significantly improved, which was closely related to the sensor parameters, and the estimatable ability of some error states was improved. In addition, a comparison with traditional methods was also carried out. The result shows that the positioning accuracy of the proposed method can be improved by at least 60.61%.

According to the existing theories and the experimental results in this paper, it can be seen that RTS smoothing technology has a significant improvement effect on the jump phenomenon of the error states. Therefore, not only the position measurement, but other excellent measurement information that can be captured may also cause the error state to jump. At this time, RTS smoothing can still play an important role, which can be further studied.

## Figures and Tables

**Table 1:** Specifications of the MIMU and initial errors.

Initial errors	Initial attitude errors (°)	[0.3;0.3;1]
	Initial velocity errors (m/s)	[0.01;0.01;0.01]
	Initial position errors (m)	[0.05;0.05;0.05]
Gyroscope	Bias repeatability (°/h)	720
	$\mathrm{Random}\;\mathrm{walk}\;({}^{\circ}/\sqrt{h}$)	0.6
Accelerometer	Bias repeatability (mg)	3
	$\mathrm{Random}\;\mathrm{walk}\;(\mathrm{mg}/\sqrt{\mathrm{Hz}}$)	0.08

**Table 2:** Error parameters to be accumulated.

	Gyroscope		Accelerometer	
	Constant Bias (°/h)	$\mathrm{Random}\;\mathrm{Walk}\;({}^{\circ}/\sqrt{h})$	Constant Bias (mg)	$\mathrm{Random}\;\mathrm{Walk}\;(\mathrm{mg}/\sqrt{\mathrm{Hz}})$
Par 1	100	0	0	0
Par 2	200	0	0	0
Par 3	300	0	0	0
Par 4	0	1	0	0
Par 5	0	2	0	0
Par 6	0	3	0	0
Par 7	0	0	1	0
Par 8	0	0	2	0
Par 9	0	0	3	0
Par 10	0	0	0	1
Par 11	0	0	0	2
Par 12	0	0	0	3

**Table 3:** SEP in each cutting cycle.

	Cutting Cycle	SEP (m)
Traditional method without compensation	A1	1.75
	A2	1.99
	A3	2.28
	A4	2.31
Traditional method with compensation	A1	0.21
	A2	0.29
	A3	0.33
	A4	0.17
Proposed method	A1	0.50
	A2	0.32
	A3	0.13
	A4	0.17

## Data Availability

Data sharing not applicable.

## Appendix A

The settings of the initial state, $x_{0}$, initial covariance matrix, $P_{0}$, and system noise variance matrix, $Q_{k}$, are the key to Kalman filter recursion. Referring to Table 1, these can be set as:

(A1) $$x_{0}=0_{19\times 1}$$

(A2) $$P_{0}=\mathrm{diag}{\left(\begin{array}{l} {}[0.01\;m/s;0.01\;m/s;0.01\;m/s;0.3{}^{\circ};0.3{}^{\circ};1{}^{\circ};0.05\;m;0.05\;m;0.05\;m;\\ 720{}^{\circ}/h;720{}^{\circ}/h;720{}^{\circ}/h;3\;\mathrm{mg};3\;\mathrm{mg};3\;\mathrm{mg};3{}^{\circ};3{}^{\circ};3{}^{\circ};0.05]\times 3 \end{array}\right)}^{2}$$

(A3) $$Q_{k}=\mathrm{diag}{\left(\begin{array}{l} {}[0.08\;\mathrm{mg}/\sqrt{\mathrm{Hz}};0.08\;\mathrm{mg}/\sqrt{\mathrm{Hz}};0.08\;\mathrm{mg}/\sqrt{\mathrm{Hz}};\\ 0.6{}^{\circ}/\sqrt{h};0.6{}^{\circ}/\sqrt{h};0.6{}^{\circ}/\sqrt{h};0_{13\times 1}]\times 5 \end{array}\right)}^{2}t_{s}$$

where $t_{s}$ is equal to the SINS solution cycle and set as 0.01 s. The units presented above are for easy interpretation, and should be changed to SI in practical application.

Note: After a period of use, MIMU is affected by many factors, and its system noise may not meet the specifications in the manual. At this time, the value of $Q_{k}$ can be appropriately amplified.
